# Is the HPA Axis as Target for Depression Outdated, or Is There a New Hope?

**DOI:** 10.3389/fpsyt.2019.00101

**Published:** 2019-02-28

**Authors:** Andreas Menke

**Affiliations:** Center of Mental Health, Department of Psychiatry, Psychosomatics and Psychotherapy, University Hospital of Wuerzburg, Wuerzburg, Germany

**Keywords:** precision medicine, personalized medicine, biomarker, depression, HPA axis, glucocorticoid receptor, CRH1, FKBP5

## Abstract

Major depressive disorder (MDD) is a very common stress-related mental disorder that carries a huge burden for affected patients and the society. It is associated with a high mortality that derives from suicidality and the development of serious medical conditions such as heart diseases, diabetes, and stroke. Although a range of effective antidepressants are available, more than 50% of the patients do not respond to the first treatment they are prescribed and around 30% fail to respond even after several treatment attempts. The heterogeneous condition of MDD, the lack of biomarkers matching patients with the right treatments and the situation that almost all available drugs are only targeting the serotonin, norepinephrine, or dopamine signaling, without regulating other potentially dysregulated systems may explain the insufficient treatment status. The hypothalamic-pituitary-adrenal (HPA) axis is one of these other systems, there is numerous and robust evidence that it is implicated in MDD and other stress-related conditions, but up to date there is no specific drug targeting HPA axis components that is approved and no test that is routinely used in the clinical setting identifying patients for such a specific treatment. Is there still hope after these many years for a breakthrough of agents targeting the HPA axis? This review will cover tests detecting altered HPA axis function and the specific treatment options such as glucocorticoid receptor (GR) antagonists, corticotropin-releasing hormone 1 (CRH_1_) receptor antagonists, tryptophan 2,3-dioxygenase (TDO) inhibitors and FK506 binding protein 5 (FKBP5) receptor antagonists.

## Introduction

With a life-time prevalence around 20% major depressive disorder (MDD) is a very common disorder. In Europe it is one of the three most disabling conditions, next to dementias and alcohol abuse (1) and the burden of disease is projected to climb (2). MDD is associated with a substantially increased mortality due to suicide and an increased risk for serious medical conditions such as heart diseases, diabetes, and stroke (3). Although a range of effective antidepressants are available, more than 50% of patients do not respond to the first antidepressant treatment they are prescribed (4) and around 30% fail to respond even after several treatment approaches (5). Of note, even remitted patients suffer from a functional impairment (6). These non-sufficient treatment options that are currently available are reflected by the high personal and societal burden with increased rates of sick leave and early retirement (1). The commonly used treatment options do not only struggle with high rates of partial or no response, but also with a delayed onset of treatment effects and uncomfortable or even threatening adverse side effects (3). Various factors may explain the current situation: MDD is a heterogeneous condition with poorly defined endophenotypes or subgroups, the currently available drugs have very similar treatment mechanisms and target almost only components of the serotonin, norepinephrine, or dopamine signaling, and there are no biomarkers to predict the response or side effects to specific interventions (7). Moreover, the diagnostic process and treatment choice are solely based on clinical experience and intuition. Fortunately, initiatives are under way to provide individualized treatment options for each patient: personalized medicine and precision medicine are employed to match individual patients with the most effective treatment options (7). Personalized and precision medicine are often used interchangeable, however, they describe two different concepts. Personalized treatment has been administered for the last decades, physicians considered sex, age, weight, co-medication together with renal, and liver functioning, comorbidities, core-symptoms (disturbances of sleep and appetite, psychotic vs. non-psychotic, agitated vs. non-agitated …) and patients preferences in the selection process of a suitable antidepressant. However, this personalization resembles a trial and error process and is highly dependent on the experience and the knowledge of the physician (8). The objective of precision medicine is to improve the selection of effective antidepressants with best possible response and minimal side effects using genetic markers or biomarkers derived from peripheral blood, imaging, neuropsychological tests, or behavioral measures (9–11). Given the high prevalence of MDD, another task of precision medicine will be the identification of individuals at risk and then to deliver specific interventions to avoid the full development of MDD (12).

## Genetic and Environmental Factors Contributing to MDD

A meta-analysis with more than 21,000 individuals observed a heritability of MDD around 40%, common environmental factors had very small effects, but individual environmental factors showed a substantial contribution of around 60% (13). In fact, the development of MDD is crucially dependent on gene x environment interactions (14–17). Aversive environmental events such as sexual, physical, or emotional childhood trauma have been robustly associated with MDD (18). But still it is not understood how early aversive events interact with genetic and epigenetic factors to confer vulnerability to MDD and how to treat patients who have experienced early life adversity. Meanwhile there is growing evidence showing that childhood trauma substantially shapes biological systems that are responsible for a fight-or-flight response, such as the hypothalamic-pituitary-adrenal (HPA) axis. In fact, childhood trauma may lead to an increased sensitivity of the HPA axis and to heightened responses to subsequent stressors (19, 20). Thus, the HPA axis may be a suitable target for specific interventions.

## HPA Axis

**Figure 1:**
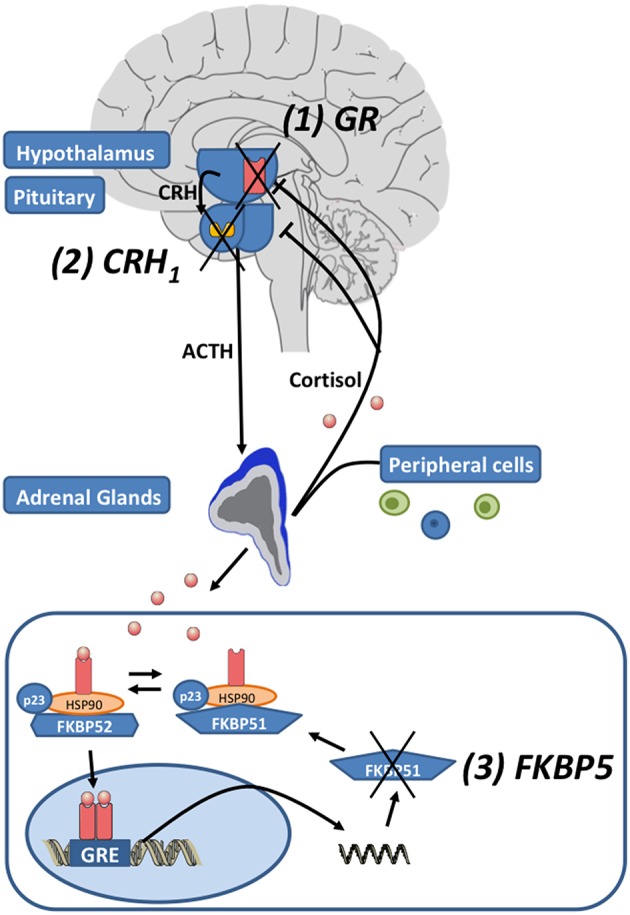
The hypothalamic-pituitary-adrenal (HPA) axis: Corticotropin-releasing hormone (CRH) is released by neurons in the paraventricular nucleus of the hypothalamus. Subsequently CRH_1_ receptors are activated and the secretion of adrenocorticotropic hormone (ACTH) from the pituitary is induced. ACTH induces the release of glucocorticoids (cortisol) by the adrenal glands. After the activation of the HPA axis, negative feedback loops are activated to reinstate homeostasis by cortisol activating glucocorticoid receptors (GR). The unliganded GR complex consists of the co-chaperones FKBP51 or FKBP52 (encoded by their respective genes *FKBP5* and *FKBP4*), p23 (a co-chaperone molecule) and hsp90 dimer. When FKBP51 binds to the GR-complex via hsp90, the GR affinity for cortisol is reduced. When glucocorticoids bind to the GR, FKBP51 is exchanged against FKBP52 and the nuclear translocation of the ligand-bound GR is enabled. The GR directly binds to the DNA via glucocorticoid response elements (GREs) and induces FKBP5 mRNA expression and subsequently FKBP51 production, inducing an ultra-short negative feedback loop on GR sensitivity. Drugs regulating the function of the HPA axis target: (1) the GR, (2) the CRH_1_ receptors, and (3) *FKBP5*/FKBP51 (modified after Leistner and Menke (21); Copyright (2018), with permission from Elsevier).

Environmental stress activates the release of the monoamines serotonin, norepinephrine and dopamine from the amygdala, hippocampus, and other brain regions. Subsequently, the paraventricular nucleus (PVN) of the hypothalamus synthesizes corticotrophin-releasing hormone (CRH), that binds to corticotropin-releasing hormone 1 (CRH_1)_ and CRH_2_ receptors in the anterior pituitary. Then ACTH is secreted in the circulation (see Figure 1). ACTH activates the production and release of glucocorticoids (GC) in the adrenal glands. To reinstate homeostasis negative feedback mechanisms are initiated: GCs bind to glucocorticoid receptors (GR) of the hippocampus, the PNV and the anterior pituitary gland and thus inhibit the further release of CRH (22). In MDD the sensitivity of the GR is impaired leading to a reduced negative feedback mechanism and subsequently to a central hypersecretion of CRH and an increased production of GCs (23, 24). The sensitivity of the GR is substantially regulated by *FKBP5*, encoding the FK 506 binding protein 51 or FKBP51, a co-chaperone of heat-shock protein 90 (hsp90) (25). When FKBP51 is bound to the GR complex, the affinity for glucocorticoid-binding is reduced and the GR is translocated into the nucleus less efficiently. *FKBP5* mRNA and protein expression are induced by GR activation and provide an ultra-short negative feedback loop for GR sensitivity (25). Polymorphisms within *FKBP5* have been shown to be associated with differential regulation of *FKBP5* mRNA expression after activation of GR and differences in GR sensitivity (26, 27). *FKBP5* has been implicated in several mental disorders and stress-related conditions such as major depression (26), bipolar disorder (28), childhood trauma and posttraumatic stress disorder (29), aggressive and suicidal behavior (30, 31). Above the cellular level these genetic variants in combination with epigenetic alterations were associated with structural and functional changes in several brain regions (32–34) and with impaired working memory and cardiac stress reactivity (35). Recently *FKBP5* has been associated with metabolic function, diabetes and obesity (36–38) and pain (39, 40).

Stress-induced cortisol excess was also observed to impact the kynurenine pathway by enhancing the hepatic activity of tryptophan 2,3-dioxygenase (TDO) (41–43). Aside indoleamine 2,3-dioxygenase (IDO) TDO is the first and rate-limiting enzyme that catalyzes the conversion of tryptophan into N-formyl-kynurenine (NFK) (44). Downstream several kynurenine pathways metabolites have been associated with the development of major depression as they exert neurotoxic effects, e.g., by activating N-methyl-D-aspartate (NMDA) receptors or enhancing free radical production (45–48).

## Tests Detecting the Function of the HPA Axis

Different tests have been developed to measure the function of the HPA axis (21). The dexamethasone suppression test [DST, (49)] identifies an impaired suppression of dexamethasone on cortisol, as observed in depressed patients (50–52). However, the DST has not reached clinical relevance as a diagnostic tool because of its low sensitivity, which ranges between 20 and 50% (52–54). To increase the sensitivity and the specificity the DST was combined with the CRH stimulation test, the dexamethasone-corticotropin-releasing hormone [dex-CRH, (55, 56)] test, which actually led to an improved sensitivity in detecting alterations of the HPA axis with a successful classification of up to 80% of depressed patients (55, 56). These findings could be replicated in several studies (57–59), but others observed negative results when analyzing case-control differences (60–62). Interestingly, in addition to its ability to identify depressed patients, several studies observed that the dex-CRH test may allow a stratification of depressed patients and predict treatment outcome and disease course (21). Studies reported an increased cortisol response to the dex-CRH test in patients after remission at risk of relapse (59, 63, 64), in subjects with violent suicide attempts and suicide completion (65) and in melancholic patients compared to non-melancholic depressed patients (66). Contrary, a reduced cortisol response in the dex-CRH test was found in depressed patients with suicidal behavior (67) and women with chronic social stressors (68). An early normalization of the dex-CRH test results has been associated with response to antidepressant medication (59). However, we observed previously, that the readouts of the dex-CRH test are substantially dependent on the plasma concentrations of dexamethasone, thus several factors that influence the plasma concentration do also impact the readout of the test (69). Recently we reported on the potential use of the dexamethasone-induced gene expression changes as an additional indicator for alterations of the HPA axis and as a potential biomarker in depression (70) and other stress-related mental disorders such as job-related exhaustion (71). For this test, before and 3 h after a GR activation by dexamethasone cortisol, ACTH, blood count, and gene expression signatures are measured to detect GR sensitivity alterations (21). Of note, this test was not dependent on dexamethasone plasma concentrations (69). Applying this test we observed an increased GR sensitivity in patients with anxious depression compared with non-anxious depression, an enhanced leukocyte reactivity in patients with childhood trauma (72) and an increased GR sensitivity in healthy women compared to healthy men (73). In a broader, stimulated expression quantitative trait locus (eQTL) approach we combined these gene expression signatures after GR-activation with genome-wide single nucleotide polymorphism (SNP) data and found that common genetic variants that modulate the transcriptional response to GR-activation mediate the risk for MDD as well as other mental disorders (74).

## Specific Targets of the HPA Axis

### GR Antagonists

Based on numerous findings of a HPA axis hyperactivity in patients with psychotic depression open-label and double-blind trials with the GR antagonist (and also progesterone antagonist) mifepristone were conducted (75) (Figure 1). The studies using dosages between 300 and 1,200 mg /d showed mixed results, with both positive studies and failed trials (75). A combined analysis of similarly designed double-blind phase 2 or 3 studies assessing the efficacy and safety of 7 day mifepristone treatment revealed a meaningful efficacy (*p* < 0.004) for mifepristone in reducing psychotic symptoms, adverse events were similar in mifepristone and placebo treated patients (76). Interestingly, high mifepristone plasma concentrations were associated with the strongest response, followed by changes in cortisol and ACTH (76). There is also accumulating evidence that mifepristone ameliorates cognitive deficits in major depression and bipolar disorder (77). Thus, for depressed patients with psychotic features a GR antagonist such as mifepristone may be an individualized treatment option.

### CRH_1_ Receptor Antagonists

In preclinical models central administration of CRH produces behavioral effects that closely resemble the symptoms of depression in humans (78, 79). These effects are attenuated by central administration of a specific CRH receptor antagonist (79, 80). Moreover, also clinical studies provided evidence of a CRH hyperactivity in depression and anxiety (79). A clinical trial using the CRH_1_ receptor antagonist R121919 in the treatment of major depression revealed significant reductions in the Hamilton Depression Rating Scale (HAMD) over the 30 day treatment period (81). The stress-elicited secretion of cortisol was reduced, however, it did not impair the CRH-induced release of ACTH and cortisol and thus the stress hormone system responsivity to CRH remained unchanged (81). However, the study did not include design components such as blinding, randomization or a placebo control and R121919 was withdrawn due to liver enzyme elevations. A further trial using another CRH_1_ receptor antagonist, CP-316,311 did not observe a significant difference between patients treated with CP-316,311 and placebo (82). Other trials using CRH_1_ receptor antagonists in patients with major depression, social and generalized anxiety disorder and suicidal ideation could also not reveal beneficial effects (83). In a trial with anxious, alcohol-dependent women the CRH_1_ receptor antagonist Verucerfont (also GSK561679) produced a dampening of the HPA axis response to social stressors and attenuated amygdala response to negative affective stimuli, while alcohol craving was unaffected (84). Recently a double-blind, randomized and placebo-controlled trial investigated the efficacy of the same CRH_1_ receptor antagonist in women suffering from Posttraumatic stress disorder (85, 86). The trial did not observe a significant improvement of PTSD symptoms in patients treated with GSK561679 compared to placebo overall (86). However, subjects with a moderate or severe history of childhood abuse and a certain CRH1 receptor SNP genotype did only response to GSK561679, not to placebo (86). Nevertheless, the authors concluded that CRH_1_ receptor antagonists as a class are ineffective as monotherapy for stress-related mental disorders (86) and the question arose whether it is time to call it quits for the CRH_1_ receptor antagonists (83, 87). CRH is a key regulator of the stress response and controls endocrine activity by direct modulation of the HPA axis. As stated above, numerous preclinical and clinical data support the involvement of CRH and CRH_1_ receptors in stress-related mental disorder (88). However, some of the tested agents did only show meaningful effects in some of the preclinical stress tests, moreover, preclinical data not always translate to clinical trials without complications (83, 87). For CRH_1_ receptor antagonists, the traditional clinical trial design is probably not suitable. Instead, patients with an overactivity of the CRH—CRH1 receptor signaling should be identified by reliable biological measures in terms of precision medicine, that is already well-established in other medical fields, such as oncology (87), and then included in a respective trial. Thus, CRH_1_ receptor antagonists are still promising agents for stress-related mental disorders, but probably only in those patients who are subject to a significant CRH signaling dysfunction.

### TDO Inhibitors

TDO inhibition by directly targeting the kynurenine production is supposed to decrease neurotoxic metabolites and thus ameliorate depressive symptoms (44). TDO inhibition is a mechanism shared by the largest number of antidepressants, e.g., citalopram effectively decreases TDO activity (44, 89). Interestingly, agents inhibiting glucocorticoids such as RU486 showed antidepressive properties by inhibiting TDO activity (90, 91). Additionally, co-treatment with allopurinol, also a TDO inhibitor, improved chronic stress induced depressive-like behavior (92). Recently, the agent NSC 36398, a flavonoid compound, was observed to be a first selective TDO inhibitor (93).

### FKBP5 Antagonists

As described above *FKBP5*, respectively, FK506 binding protein 51/FKBP51 regulates the responsiveness of the GR and the HPA axis and is also implicated in important gene x environment interactions underlying stress-related mental disorders (25, 94), making it a promising drug target. In fact, several research groups have consistently observed protective effects of *FKBP5* knock-out or knock-down on stress-coping behavior and stress endocrinology in preclinical models of depression and anxiety (95). The prototypic FKBP ligand FK506 and rapamycin showed the principal druggability. In addition, FKBP51 is highly suited for X-ray cocrystallography, which facilitates the rational drug design (96). Sulfonamide analogs have been found that possess FKBP51 binding properties (97). However, drug discovery has been hampered by the inability that all known ligands cannot differentiate FKBP51 and the opposing homolog FKBP52 (98, 99). Recently with SAFit1 and SAFit2 two promising potent and highly selective inhibitors of FKBP51 were discovered, that achieved selectivity by an induced-fit mechanism and improved neuroendocrine feedback and stress-coping behavior in mice (100, 101). Of note, co-application of SAFit2 with the selective serotonin reuptake inhibitor escitalopram, a common antidepressant, lowered the efficacy of escitalopram in anxiety-related tests but improved stress coping behavior in a mouse model (102). *FKBP5* antagonists may be promising new treatment options for patients suffering from stress-related mental disorders and who have an altered functioning of *FKBP5*/GR/HPA axis signaling.

## Conclusion

Despite the very strong preclinical and clinical data of a dysregulation of the HPA axis in stress-related mental disorders, such as major depression, no drug has been approved that targets specific components of the HPA axis. In addition, no test is routinely used in the clinical setting to identify patients with a measureable HPA axis dysfunction. In fact, there is evidence that not all depressed patients display alterations of the HPA axis, and therefore not all of them would benefit of a very specific treatment, targeting only HPA axis components. This has become abundantly clear with the failing CRH_1_ receptor antagonists for major depression and posttraumatic stress disorder. However, even in the failed trials, there were initial hints that subgroups of patients carrying certain genetic risk variants or having a history of childhood trauma would indeed benefit from these very specific treatment options (86). Therefore, precision medicine has to be employed to match the specific antidepressant agent to the specific underlying biological alteration and the individual patient. Biological markers derived from tests detecting the HPA axis function, GR sensitivity, or *FKBP5* dysregulation are necessary to identify suitable patients for these specific agents. In addition, clinical variables such as psychotic symptoms or history of childhood trauma combined with certain genetic risk variants may further improve the accuracy of such a test. Still in its infancy, the dexamethasone-induced gene expression test may become a promising tool to assess the GR sensitivity and FKBP5 function (21), because it combines neuroendocrine results with molecular genetic patterns of a GR challenged gene expression integrating genetic risk polymorphisms and additional clinical data (21). Thus, the future algorithms defining the treatment of major depression or other stress-related mental disorders will incorporate tests that stratify patients groups and match individual patients with highly specific agents, for example that target HPA axis components such as the GR, CRH_1_ receptors or *FKBP5*. GR antagonists, especially mifepristone, have provided very promising results for the treatment of psychotic depression so far and therefore could gain more relevance (76). CRH_1_ receptor antagonists have experienced a setback, but after employing suitable tests to find susceptible patients this development could be reversed. FKBP5, representing a molecular hub modulating many cellular pathways, is a novel and very promising candidate to target component of the stress hormone system and to ameliorate stress-related mental disorders and other sequelae of stress.

## Author Contributions

The author confirms being the sole contributor of this work and has approved it for publication.

### Conflict of Interest Statement

The author declares that the research was conducted in the absence of any commercial or financial relationships that could be construed as a potential conflict of interest.
